# *SND1*作为肺腺癌癌基因和预后标志物的鉴定与分析

**DOI:** 10.3779/j.issn.1009-3419.2023.102.47

**Published:** 2024-01-20

**Authors:** Ruihao ZHANG, Hua HUANG, Guangsheng ZHU, Di WU, Chen CHEN, Peijun CAO, Chen DING, Hongyu LIU, Jun CHEN, Yongwen LI

**Affiliations:** ^1^300052 天津，天津医科大学总医院肺部肿瘤外科（张芮豪，黄华，朱光胜，吴迪，曹培俊，陈军）; ^1^Department of Lung Cancer Surgery, Tianjin Medical University General Hospital; ^2^天津市肺癌研究所，天津市肺癌转移与肿瘤微环境重点实验室（陈琛，刘红雨，陈军，李永文）; ^2^Tianjin Key Laboratory of Lung Cancer Metastasis and Tumor Microenvironment, Tianjin Lung Cancer Institute, Tianjin Medical University General Hospital, Tianjin 300052, China; ^3^832003 石河子，石河子大学医学院第一附属医院胸外科（丁晨，陈军）; ^3^Department of Thoracic Surgery, First Affiliated Hospital, School of Medicine, Shihezi University, Shihezi 832003, China

**Keywords:** SND1, 肺肿瘤, 细胞周期, 免疫浸润, SND1, Lung neoplasms, Cell cycle, Immune infiltration

## Abstract

**背景与目的** 转录因子（transcription factor, TF）可以结合特异性序列，对下游基因起到促进或抑制的作用，对肿瘤的发生、迁移、侵袭等生物学过程都有重要的影响。葡萄球菌含核酸酶结构域1（Staphylococcal nuclease-containing structural domain 1, SND1）作为一种转录共激活因子，被认为是肿瘤治疗的一个潜在靶点。然而，其在肺腺癌（lung adenocarcinoma, LUAD）中的表达及作用尚不清楚。本研究主要探索SND1作为LUAD癌基因在LUAD中的作用。**方法** 从癌症基因组图谱（The Cancer Genome Atlas, TCGA）、基因表达综合数据库（Gene Expression Omnibus, GEO）、临床蛋白质组肿瘤分析联盟（Clinical Proteomic Tumor Analysis Consortium, CPTAC）和人类蛋白图库（Human Protein Atlas, HPA）等数据库获取数据，探讨SND1表达与LUAD患者预后、免疫细胞浸润和亚细胞定位的关系；利用EdU法、CCK-8法、流式细胞术、细胞划痕实验、Transwell实验、蛋白印迹实验等体外实验探讨SND1在LUAD中的功能作用。**结果** 在LUAD中SND1的表达上调，且与患者预后不良有关。SND1主要存在于LUAD细胞的细胞质中。富集分析表明SND1与细胞周期密切相关，包括DNA复制和染色体分离等。免疫细胞浸润分析显示，SND1与多种免疫细胞群有关，包括T细胞、B细胞、细胞毒性细胞和树突状细胞。体外研究表明沉默SND1可抑制LUAD细胞的增殖、侵袭和迁移，下调SND1可阻断G_1_期细胞周期进程。**结论** SND1可能是LUAD重要的预后生物标志物，其可促进LUAD细胞的增殖和迁移。

## Abstract (en)

**Background and objective** Transcription factor (TF) can bind specific sequences that either promotes or represses the transcription of target genes, and exerts important effects on tumorigenesis, migration, invasion. Staphylococcal nuclease-containing structural domain 1 (SND1), which is a transcriptional co-activator, is considered as a promising target for tumor therapy. However, its role in lung adenocarcinoma (LUAD) remains unclear. This study aims to explore the role of SND1 in LUAD. **Methods** Data from The Cancer Genome Atlas (TCGA), Gene Expression Omnibus (GEO), Clinical Proteomic Tumor Analysis Consortium (CPTAC), and Human Protein Atlas (HPA) database was obtained to explore the association between SND1 and the prognosis, as well as the immune cell infiltration, and subcellular localization in LUAD tissues. Furthermore, the functional role of SND1 in LUAD was verified in vitro. EdU assay, CCK-8 assay, flow cytometry, scratch assay, Transwell assay and Western blot were performed. **Results** SND1 was found to be upregulated and high expression of SND1 is correlated with poor prognosis of LUAD patients. In addition, SND1 was predominantly present in the cytoplasm of LUAD cells. Enrichment analysis showed that SND1 was closely associated with the cell cycle, as well as DNA replication, and chromosome segregation. Immune infiltration analysis showed that SND1 was closely associated with various immune cell populations, including T cells, B cells, cytotoxic cells and dendritic cells. In vitro studies demonstrated that silencing of SND1 inhibited cell proliferation, invasion and migration of LUAD cells. Besides, cell cycle was blocked at G_1_ phase by down-regulating SND1. **Conclusion** SND1 might be an important prognostic biomarker of LUAD and may promote LUAD cells proliferation and migration.

肺癌是世界上最常见的癌症之一，也是癌症死亡的重要原因^[1]^。2020年全球癌症统计数据分析^[2]^显示，肺癌导致的死亡人数约为180万，占所有癌症相关死亡人数的18.0%。肺癌根据组织病理类型可分为小细胞肺癌（small cell lung cancer, SCLC）和非小细胞肺癌（non-small cell lung cancer, NSCLC）两大组织类型。NSCLC占肺癌的85%，而肺腺癌（lung adenocarcinoma, LUAD）是NSCLC病例中的主要亚型^[3]^。近年来，尽管手术、靶向治疗和免疫治疗等各个方面取得了重大进展，但LUAD患者的5年生存率仍低于15%^[4]^。因此，鉴定LUAD的诊断、治疗和预后预测的新靶点至关重要。

葡萄球菌含核酸酶结构域1（Staphylococcal nuclease and tudor domain containing 1, SND1）是一种转录共激活因子，编码SND1蛋白质，在mRNA剪接、编辑和蛋白稳定性等多种转录后调控过程中发挥重要作用。SND1在哺乳动物中普遍表达，在进化上高度保守，在多种生物学过程，包括细胞增殖、分化和凋亡中具有重要的生理作用^[5]^。SND1通过调节转录因子、信号通路、细胞周期调节因子等关键分子，促进肿瘤细胞的生长和存活。其在肺癌、肝细胞癌、乳腺癌、宫颈癌和卵巢癌等多种实体肿瘤中表达上调，通过影响肿瘤的增殖、迁移和侵袭等作用，导致患者预后不良^[6⇓⇓⇓-10]^。因此，SND1被认为是一个潜在的治疗靶点。然而，SND1在肺癌中的作用还未完全明确，需要进一步的研究来充分了解SND1在肺癌尤其是LUAD中的作用。

在本研究中，我们利用公共数据库研究了SND1在LUAD中肿瘤组织与相应癌旁组织中的表达差异，分析SND1表达与LUAD患者预后的关系以及LUAD中SND1表达与免疫细胞浸润的相关性；利用体外实验探讨SND1对LUAD细胞生物学行为的影响。我们的研究进一步探索了SND1在LUAD中的潜在致癌作用，证实SND1可能是LUAD诊断和治疗的潜在生物标志物和治疗靶点。

## 1 资料与方法

### 1.1 数据来源和生物信息分析

分别从基因表达综合数据库（Gene Expression Omnibus, GEO）（https://www.ncbi.nlm.nih.gov/geo/）和癌症基因组图谱（The Cancer Genome Atlas, TCGA）（https://portal.gdc.cancer.gov/）中获取与LUAD相关的基因数据，共包含3个数据集，分别是TCGA-LUAD数据集以及2个GEO数据集（GSE116959和GSE68517）。其中TCGA-LUAD数据集包含510例LUAD患者的转录组数据，而GSE116959和GSE68517两个数据集分别包含57例和86例LUAD患者的数据样本。使用人类蛋白质表达图谱（The Human Protein Atlas, HPA）分析SND1在LUAD患者中的免疫组化染色结果。利用UALCAN数据库（The University of ALabama at Birmingham Cancer Data Analysis Portal）（http://ualcan.path.uab.edu/）进行广泛的交互式生物信息学分析，包括临床因子、RNA-seq和预后相关数据。此外，从癌细胞系百科全书数据库（Cancer Cell Line Encyclopedia, CCLE）（https://sites.broadinstitute.org/ccle）获取SND1在20种LUAD细胞系的mRNA表达数据。另外，我们使用癌症基因组门户网站（c-BioPortal）分析SND1在LUAD细胞中的基因突变，并使用Kaplan-Meier曲线评估SND1的表达与肺LUAD患者总生存期（overall survival, OS）的关系。

### 1.2 功能富集分析

通过使用R中的“ClusterProfiler”软件包进行基因本体（gene ontology, GO）功能分析和京都基因和基因组百科全书（Kyoto Encyclopedia of Genes and Genomes, KEGG）通路富集分析。

### 1.3 免疫浸润分析

采用单样本基因集富集分析（single sample gene set enrichment analysis, ssGSEA）进行肿瘤免疫浸润分析，Spearman相关分析评估SND1高表达组和低表达组与24个免疫细胞浸润水平的相关性。

### 1.4 临床样本

收集天津医科大学总医院肺部肿瘤外科手术切除的LUAD组织及其对应的癌旁组织10例。本研究严格遵守天津医科大学总医院伦理委员会的伦理准则，并获得了参与者的书面同意。

### 1.5 细胞培养

肺癌细胞系（NCI-H1975、A549、NCI-H1299、HCC827和PC-9）和人正常肺上皮细胞系（BEAS-2B）均用含10%胎牛血清（Gibco, NY, USA）的DMEM培养基，于37 ^o^C、5% CO_2_浓度的培养箱中培养。

### 1.6 实时荧光定量逆转录聚合酶链反应（quantitative real-time polymerase chain reaction, qRT-PCR）

常规Trizol法提取细胞总RNA，2.0 μg总RNA用反转录试剂盒PrimeScript^TM^ RT reagent Kit[TaKaRa，宝生物工程（大连）有限公司]反转录成cDNA，然后按照产品说明书进行qRT-PCR。实时荧光定量PCR检测SND1和GAPDH mRNA水平。SND1引物序列为：正向引物5’-GGTGGACTACATTAGACCAGCC-3’，反向引物5’- AGACCTTTGCTGACAAGCCTC-3’。GAPDH引物序列为：正向引物5’-GGAGCGAGATCCCTCCAAAAT-3’，反向引物5’-GGCTGTTGTCATACTTCTCATGG-3’。每个反应重复3次，以确保数据的准确性和可靠性，2^∆∆CT^法计算SND1表达水平。

### 1.7 免疫印迹

使用RIPA裂解缓冲液（上海碧云天生物技术有限公司）裂解细胞提取总蛋白。30 μg蛋白样本经10% SDS-PAGE凝胶电泳，并转移至PVDF膜上。用5% BSA室温封闭2 h。封闭后，将膜与SND1抗体（ab65078, Abcam, 1:1000）在4 °C下孵育过夜。然后在膜上孵育过氧化氢酶（horseradish peroxidase, HRP）结合的二抗2 h，曝光显影。用Image J分析条带灰度值。

### 1.8 免疫荧光

将H1975和A549细胞以每孔1×10^5^个接种于无菌玻片上，培养24 h后取出玻片并用4%多聚甲醛溶液固定，以保持其形状和结构的完整性。细胞经用0.1% Triton X-100处理后，5% BSA封闭液，加入SND1（1:200）抗体4 °C孵育过夜。随后，加入荧光标记的二抗于37 °C孵育40 min。荧光显微镜下观察SND1的表达和亚细胞定位。

### 1.9 细胞周期分析

收集并清洗H1975和A549细胞沉淀，70%乙醇4 °C固定过夜，经RNase A在37 °C下孵育30 min处理，终浓度50 μg/mL的碘化丙啶（propidium iodide, PI）室温避光孵育30 min。使用流式细胞仪对细胞周期进行检测。

### 1.10 免疫组化（immunohistochemistry, IHC）

常规脱蜡法处理患者病理切片，柠檬酸钠缓冲液进行抗原修复，SND1抗体（1:100）4 °C孵育过夜。经PBS冲洗3次后，与二抗室温孵育1 h再滴加新鲜的DAB进行染色，经苏木素复染，并进行镜检分析。

### 1.11 细胞增殖分析

EdU实验用来评估细胞增殖情况。细胞以每孔6×10^3^个的密度接种于96孔板上，培养箱中培养48 h。每孔中加入10 μL EdU试剂孵育2 h。细胞经4%多聚甲醛和0.5% Triton X-100溶液固定和透化处理后，Hoechst染色并通过荧光显微镜拍照记录。通过公式：EdU阳性细胞（红色染色）数/Hoechst 33342阳性细胞（蓝色染色）总数，计算分析细胞的增殖情况。CCK-8法分析细胞增殖。收集细胞，以每孔1×10^4^个细胞接种于96孔板上，细胞培养0、12、24、48 h后加入CCK-8处理，酶标仪检测光密度（optical delnsity, OD）值后分析细胞增殖情况。所有实验均重复3次，以确保统计分析的可信度。

### 1.12 侵袭试验

Transwell小室实验用来评估细胞侵袭情况。Transwell小室预先用基质凝胶（Corning）包被过夜。将1.2×10^5^个细胞以100 μL不含胎牛血清的培养基接种于上室，下室中加入600 μL含有10% FBS的完全培养基作为趋化诱导剂。孵育48 h后，用4%的多聚甲醛固定，并用0.1%的结晶紫染色。显微镜下拍照，分析细胞的侵袭情况。每项实验均独立进行3次。

### 1.13 统计分析

使用GraphPad Prism 9.0软件进行统计分析。计量数据用均数±标准差（Mean±SD）表示。采用t检验来评估两组间的差异，P<0.05表示差异具有统计学意义。

## 2 结果

### 2.1 SND1在LUAD中的表达上调

为了探索人源SND1在LUAD中的致癌作用，我们首先利用cBioPortal数据库分析了SND1在LUAD中的突变情况，结果显示SND1基因在超过3%的LUAD患者中存在基因改变，主要表现为突变和深度缺失（图1A）。通过在TCGA和GEO数据库收集的数据集分析SND1在LUAD中的表达情况，提示LUAD组织的SND1 mRNA表达量明显高于癌旁肺组织（P<0.001）（图1B）。进一步，通过分析TCGA数据库中57例LUAD组织和其配对远癌组织，证实了SND1在LUAD组织中显著上调（图1C）。GEO数据集GSE116959和GSE68517的分析也得出相似的结果（图1D）。

**图 1:**
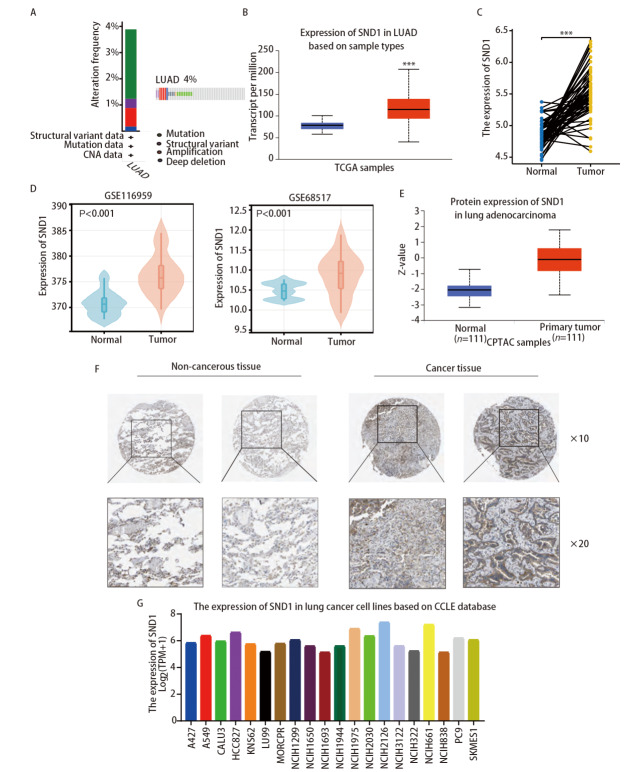
SND1在LUAD中的表达的情况。 A：基于TCGA的LUAD数据库，通过cBioPortal分析SND1的基因变化突变情况；B：TCGA数据库（UCLCAN）中LUAD组织中SND1 mRNA表达高于癌旁组织；C：SND1在配对肿瘤和正常组织中的表达；D：经GEO中两个数据库（GSE116959和GSE68517）验证，SND1在肿瘤组织中高表达；E：在CPTAC数据库中，SND1蛋白在LUAD组织中高表达；F：在人类蛋白质图谱数据库中免疫组化检测SND1在LUAD组织和癌旁肺组织中的蛋白表达；G：CCLE数据库中20个NSCLC细胞系中SND1的表达情况。

为了进一步明确SND1在LUAD中的表达情况，我们使用CPTAC数据库分析SND1在LUAD中的蛋白表达情况，结果显示，LUAD组织中的SND1蛋白表达相较癌旁肺组织显著升高（图1E）。此外，HPA数据库中的分析结果也表明，与配对的远癌组织相比，SND1在LUAD组织中的蛋白表达显著升高（图1F）。为了进一步验证研究结果，我们利用CCLE数据集来分析SND1基因在各NSCLC细胞系中的表达情况，结果显示，SND1在多个肺癌细胞系中的表达显著上调（图1G）。

### 2.2 SND1表达与LUAD患者临床因素的相关性

为了探讨SND1的表达和患者临床病理特征的关系，我们利用UALCAN数据库分析SND1在不同肿瘤分期和淋巴结转移不同阶段的表达情况。结果表明，在肿瘤分期I和II期及淋巴结转移阴性的患者组织中，SND1的表达水平较低（图2A、2B）。而且，SND1蛋白表达与LUAD的分化程度密切相关，分化程度越高，SND1蛋白越高（图2C）。采用Kaplan-Meier评估SND1表达与生存时间的相关性。结果提示，SND1高表达患者的OS时间明显短于SND1低表达患者（HR=1.18, 95%CI: 1.04-1.35, P=0.013）（图2D）。受试者工作特征（receiver operating characteristic, ROC）曲线分析表明SND1对LUAD具有明显的诊断价值，是LUAD重要的诊断性ROC独立指标[曲线下面积（area under the curve, AUC）为0.877]。上述结果表明SND1是LUAD重要的促癌基因，并且有可能成为LUAD的分子标志物和治疗靶点。

**图 2:**
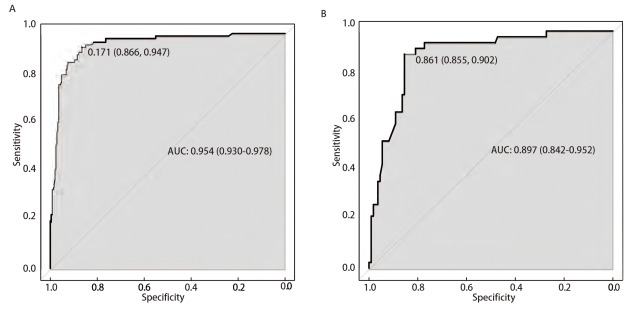
SND1与LUAD的临床相关性。 A、B：TCGA数据库（UCLCAN）中不同肿瘤分期和淋巴结转移的LUAD中SND1的表达；C：CPTAC数据库中不同分化状态LUAD中SND1蛋白表达水平；D：根据SND1在LUAD中的表达水平计算的OS的Kaplan-Meier生存曲线；E：基于TCGA数据库的SND1在LUAD中的ROC曲线。与正常对照组相比，*P<0.05，***P<0.001。

### 2.3 SND1的分布和亚细胞定位

我们通过HPA数据库分析SND1的分布和亚细胞定位，图3A显示了SND1在人不同器官中的表达图谱，可见SND1在所有器官中都有表达（图3A）。HPA数据库的细胞结构图还显示，SND1在小鼠细胞中主要定位于细胞质中（图3B）。为了进一步确定SND1在人LUAD细胞中的亚细胞定位，我们使用免疫荧光检测SND1在BEAS-2B、H1975、A549和HCC827细胞的蛋白表达情况。图3C显示SND1主要定位于LUAD细胞质中，与HPA数据库分析的数据相一致。

**图 3:**
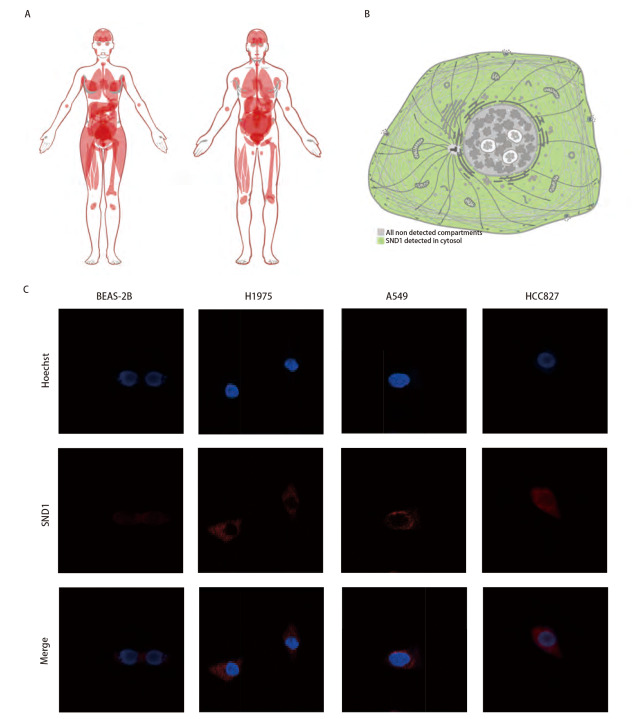
SND1在体内和细胞中的分布。 A、B：基于人类蛋白质图谱的SND1体内和细胞内分布；C：共聚焦荧光显微镜观察SND1在LUAD细胞中的表达及定位（×100）。

### 2.4 相关基因和差异基因的功能富集

为了探索SND1在LUAD中的功能，我们利用STRING数据库分析了与SND1相互作用的蛋白，并构建PPI网络图（图4A）。另外，基于TCGA的数据，我们利用Spearman相关性分析筛选出了与SND1相关性最强的500个基因。随后对这500个基因进行了KEGG通路富集和GO功能富集，结果显示，SND1参与了真核生物的内质网蛋白加工、细胞周期、细胞凋亡、DNA 复制和核糖体生物发生等生物过程和信号通路（图4B）。GO细胞成分分析结果显示SND1相关基因主要富集在细胞核中，包括细胞核、核腔、蛋白复合物、核质和细胞内无膜结构的细胞器（图4C）。GO生物过程分析结果显示，SND1相关基因主要富集在大分子生物合成过程、细胞氮化合物生物合成过程、细胞大分子生物合成过程和细胞周期（图4D）。而GO分子功能主要富集在核酸结合、RNA 结合、小分子结合、核苷酸结合和核苷磷酸结合方面（图4E）。

**图 4:**
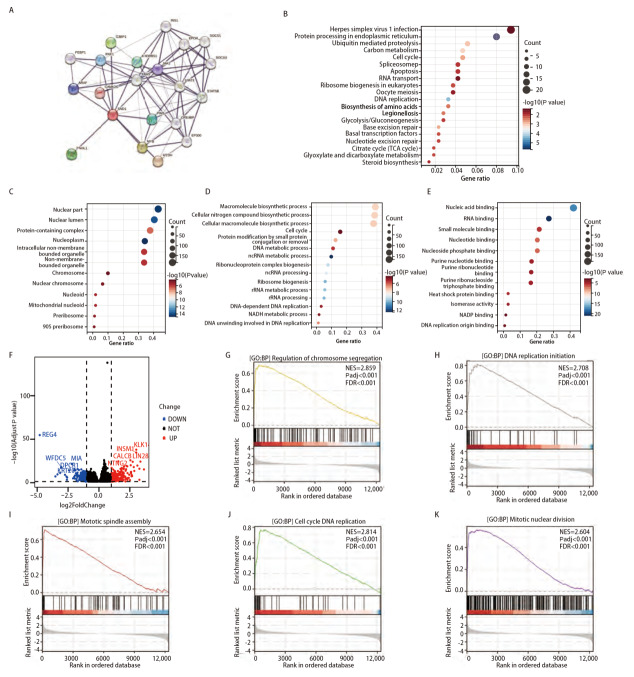
SND1相关基因的功能富集。 A：SND1相关蛋白相互作用网络；B：相关基因的KEGG富集注释；C-E：相关基因的GO富集注释，包括CC、BP和MF；F：高SND1表达组与低SND1表达组DEGs的火山图；G：染色体分离调控；H：DNA复制启动；I：有丝分裂纺锤体的组装；J：细胞周期DNA复制；K：有丝分裂核分裂。

为了进一步研究SND1的生物学功能，基于TCGA数据库中SND1的表达量将LUAD中SND1表达分为高表达组和低表达组。以调整后的P<0.05和|log2 FC|>1为临界值，我们筛选出477个差异表达基因（differentially expressed genes, DEGs）（图4F）。通过基因组富集分析（gene set enrichment analysis, GSEA），我们发现SND1差异基因在染色体分离调控基因集、DNA复制启动基因集、有丝分裂纺锤体组装基因集、细胞周期DNA复制基因集和有丝分裂核分裂基因集等重要过程的基因集中显著富集（图4G-4K）。这些发现表明，SND1可能通过影响LUAD细胞的细胞周期和染色体分离等生物学过程影响LUAD的发生发展。

### 2.5 免疫细胞浸润与LUAD中SND1的异常表达有关

为了研究SND1表达与免疫细胞浸润水平的关系，我们利用ssGSEA法进行免疫浸润分析。结果显示，16种免疫细胞类型在SND1高表达和低表达两组中的表达模式差异较大。值得注意的是，SND1高表达组的B细胞、T细胞、树突状细胞、嗜酸性粒细胞、肥大细胞和巨噬细胞的表达水平较低（图5A）。

**图 5:**
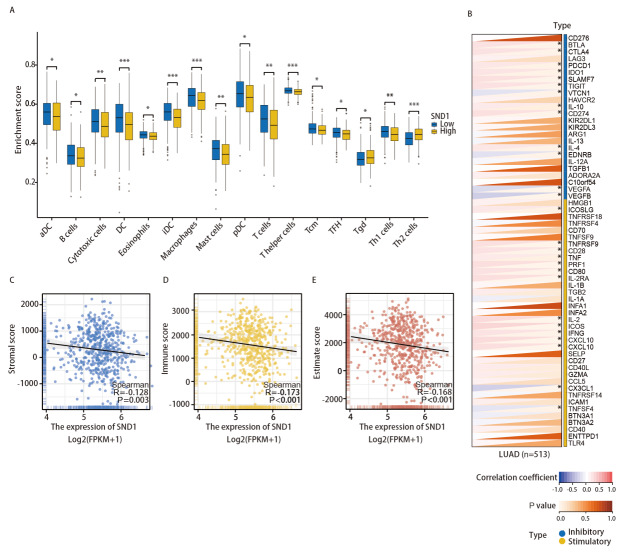
SND1表达与免疫细胞浸润。 A：SND1高表达组和SND1低表达组中16种免疫细胞的差异表达；B：SND1与LUAD中免疫检查点基因的关系；C-E：SND1表达与基质评分、免疫评分和估计评分的关系。

同时，我们还观察到SND1的表达与免疫抑制相关基因（包括BTLA、CTLA4、PDCD1、IDO1、SLAMF7、TIGIT、IL-10、CD274和IL-4）呈正相关，而与VTCN1、EDNRB、VEGFA、VEGFB则呈负相关（图5B）。此外，SND1的表达与免疫刺激相关基因，如ICOSLG、TNFRSF9、CD28、TNF、PRF1、CD80、IL-2RA、IL-2、ICOS、IFNG、CXCL10和CXCL9等呈正相关，而与CX3CL1和TNFSF4呈负相关。进一步地，通过Spearman分析，我们还发现SND1的表达与免疫反应相关的评分（包括免疫评分、估计评分和间质评分）呈负相关（图5C-5E）。这些发现共同表明，SND1与多种免疫细胞浸润和免疫相关基因存在一定联系，可能作为LUAD免疫疗法疗效潜在的预后指标。

### 2.6 SND1对LUAD细胞周期、增殖、迁移和侵袭的影响

为了验证上述的研究结果，我们检测了SND1在人正常支气管上皮细胞BEAS-2B以及H1975、A549、PC9、H1299等LUAD细胞系中的表达。与人正常支气管上皮细胞BEAS-2B相比较，SND1在4株LUAD细胞系中的mRNA和蛋白水平上的表达均显著上调（图6A、6B）。此外，免疫组化的结果显示SND1在LUAD组织中的蛋白表达明显高于其相应的癌旁组织（图6C）。

**图 6:**
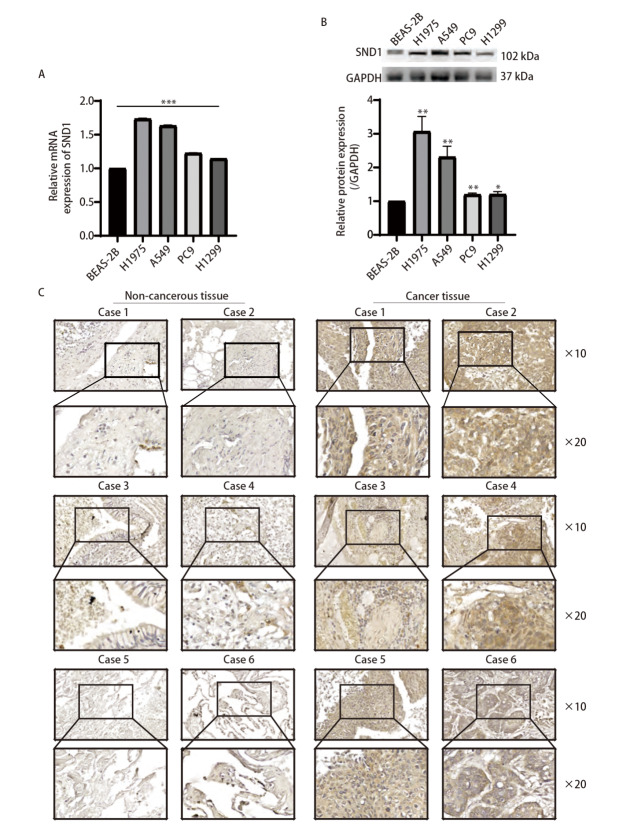
SND1在LUAD细胞系和组织中的表达情况。 A、B：SND1在支气管上皮细胞系（BEAS-2B）和4种NSCLC细胞系（H1975、A549、PC9和H1299）中的mRNA水平和蛋白水平表达；C：10例LUAD患者肿瘤和非肿瘤组织中SND1的免疫组化染色。与BEAS-2B细胞系相比，*P<0.05, **P<0.01, ***P<0.001。

为了进一步验证我们之前的发现，我们选择SND1表达量较高的LUAD细胞系H1975和A549进行下一步功能实验的探索，并利用siRNA技术抑制细胞中SND1的表达。结果显示，H1975和A549细胞转染SND1 siRNA后，SND1在mRNA和蛋白水平的表达上都出现了显著降低（图7A）。CCK-8细胞增殖实验结果显示，下调SND1表达，能明显抑制H1975和A549的细胞活力（图7B）。EdU法检测也提示敲低SND1表达能显著抑制肺癌细胞的增殖（图7C）。同时，流式细胞术检测结果显示，与H1975 NC组相比，H1975 si-SND1转染组细胞处于G_1_期的比例明显增加。在A549细胞中也得到了相似的结果（图7D、7E）。为了探索SND1对H1975和A549细胞迁移和侵袭的影响，我们进行了细胞划痕实验和Transwell实验，结果表明下调SND1表达能显著降低H1975和A549细胞的迁移和侵袭能力（图7F、7G）。为了进一步探讨SND1促进LUAD细胞增殖和迁移侵袭的机制，我们利用蛋白免疫印迹检测抑制SND1表达对细胞周期和迁移侵袭相关蛋白的影响，结果显示，抑制SND1表达能显著降低两个关键细胞周期调控因子CDK4和cyclin-D1的表达。而细胞周期抑制因子p21和肿瘤抑癌基因p53的表达显著上调，Snail和Vimentin的表达在敲低后降低（图7H）。以上结果表明，SND1在促进LUAD细胞的增殖、迁移和侵袭中发挥着重要作用。

**图 7:**
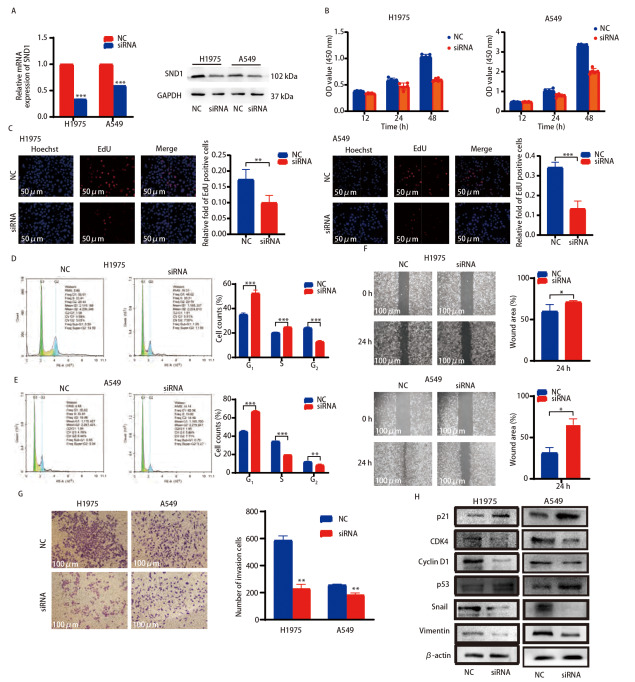
SND1对LUAD细胞周期、增殖、迁移和侵袭的影响。 A：转染si-NC和si-SND1后H1975和A549细胞的相对mRNA和蛋白水平；B：CCK-8检测敲低SND1后H1975和A549细胞的细胞增殖；C：敲低SND1后H1975和A549细胞的EdU检测结果（×20）；D、E：细胞周期检测显示SND1敲低诱导肺癌细胞G_1_期阻滞；F、G：划痕和侵袭实验结果显示，与对照细胞相比，SND1敲低细胞的迁移和侵袭能力降低（×10）；H：与对照组相比，SND1敲低组中细胞周期、迁移和侵袭相关蛋白的表达。

## 3 讨论

近年来，尽管科研人员对LUAD的治疗和潜在机制开展了大量的研究，但患者的总体生存率仍然很低^[3]^。这些患者即使接受了放疗和化疗，肿瘤仍会复发和转移，因此，迫切需要找到并验证能准确评估LUAD进展的新型预后标志物。

以往的研究^[11⇓-13]^显示，SND1在乳腺癌、直肠癌和肝细胞癌等多种实体肿瘤表达上调。同时，其表达与多种癌症的增殖、侵袭和迁移等恶性生物学过程密切相关。有研究^[14,15]^发现SND1在调节包括ERK和NF-κB在内的重要信号通路中起着至关重要的作用，而这些信号通路在肿瘤细胞的生长、转移和肿瘤耐药性中发挥重要作用。在结肠癌中，SND1阳性表达是结肠癌预后不良的独立预测因子。SND1与星形胶质细胞升高基因1（metadherin, MTDH）相互作用促进了肿瘤的发生发展^[16]^。Yu等^[17]^报道，在乳腺癌中，SND1作为转化生长因子β1（transforming growth factor-β1, TGFβ1）信号通路的一个关键调节因子，在促进乳腺癌细胞转移中发挥重要作用。然而，SND1在LUAD中的作用尚未完全清楚。因此，本研究旨在探讨SND1对LUAD细胞生物学功能和预后的影响，为LUAD的诊断治疗提供潜在靶点。我们的研究证实，SND1在LUAD肿瘤组织中的表达明显上调，SND1的高表达与LUAD临床分期和淋巴结转移密切相关。此外，对10例LUAD患者组织样本的免疫组化分析表明，与癌旁组织相比，LUAD组织中SND1的表达显著上升。同时，我们发现SND1的表达水平与LUAD细胞的增殖、迁移和侵袭能力之间存在联系。在肺癌细胞系中利用siRNA技术敲低SND1表达，周期蛋白CDK4和cyclin-D1的表达也同时下降。我们的研究结果表明SND1可能是一个癌基因，在LUAD的发生发展中起重要作用。

此外，我们还观察到SND1的表达与免疫细胞浸润水平之间存在相关性，这表明了它在肿瘤微环境中的重要性。肿瘤微环境中免疫细胞与肿瘤细胞之间的相互作用，受到免疫细胞渗入肿瘤程度的影响^[18]^。研究^[19,20]^证实了CD4^+^ T、辅助细胞（T helper, Th）和B细胞在癌症的发生发展和癌症免疫疗法中的关键作用。在乳腺癌中，SND1与MTDH形成复合物，并通过结合和破坏Tap1/2 mRNA发挥作用。这种破坏会降低肿瘤抗原的表达，从而阻碍T细胞的浸润和活化，进而使得癌细胞获得免疫逃逸^[21]^。在LUAD的研究中，B细胞和CD4^+^滤泡辅助细胞共同增强CD8^+^ T细胞的效应功能，从而加强抗肿瘤免疫反应^[22]^。此外，高水平的肥大细胞浸润和CCR2^+^细胞毒性T淋巴细胞（cytotoxic T lymphocytes, CTLs）的增加与LUAD患者的良好预后密切相关^[23]^。我们通过ssGSEA免疫浸润分析发现，SND1表达改变时也会引起免疫细胞如B细胞、T细胞、树突状细胞、嗜酸性粒细胞、肥大细胞和巨噬细胞水平的降低。免疫微环境中效应细胞比例的降低往往提示着患者的预后较差。这表明，SND1可能在调节免疫细胞的浸润中发挥重要作用，可能通过影响免疫细胞浸润程度而影响LUAD患者的预后。尽管我们的研究结果表明SND1与免疫浸润相关，然而，SND1是否可作为LUAD免疫治疗反应的标志物还需要更深入的探索，SND1影响LUAD免疫浸润的确切机制还需要更深入的研究。

综上所述，尽管本研究通过公共数据库和体外实验证实了SND1在LUAD中的重要促癌作用，为SND1作为LUAD患者重要的预后标志物提供了有价值的数据，但仍存在一些局限性。首先，我们的研究仅侧重于探讨SND1与LUAD之间的相关性，SND1参与LUAD生物学功能的具体机制尚不清楚，需要进一步的研究。其次，虽然我们进行了细胞实验验证了生物信息学的结果，但没有用动物实验进一步验证。SND1与免疫浸润细胞的相关性仅在生物信息学中得到验证，考虑到肿瘤免疫微环境的复杂性，动物模型可以更全面地了解SND1在LUAD免疫微环境中的作用。总之，本研究探讨了SND1的表达及其对LUAD恶性生物学行为以及患者预后的影响，这有助于进一步了解SND1在包括LUAD在内的人类肿瘤中的关键作用。
